# Hospital and Institutional News

**Published:** 1916-08-12

**Authors:** 


### HOSPITAL AND INSTITUTIONAL NEWS.

### A RED-CROSS KITCHENER SOUVENIR.

The Headquarters Collection Committee of the British Red Cross Society and the Order of St. John have joined hands with the Lord Kitchener National Memorial Fund in an enterprise the profits of which will be equally divided between these two patriotic organisations. This is the production and sale of facsimiles of Lord Kitchener's well-known letter; the bidding for the original produced a large sum of money, and it looks as if the sale of these reproductions should bring in even larger funds. The facsimiles are being published by Messrs. Raphael Tuck and Sons, entirely without any profit to themselves. There are three editions: one in a khaki cover at the popular price of a shilling; a slightly larger one in a white cover at half a crown; and a photogravure reproduction, which can be had for half a guinea, or in publishers' proof at a guinea and in artist's proof at two guineas. The cover is designed by Mr. Frank Brangwyn, A.R.A., and the letter is accompanied by a monograph on Lord Kitchener's career by Sir A. Conan Doyle, and by a tribute from Lord Kitchener's private secretary and intimate friend, Sir George Arthur.

### CITY OF LONDON LYING-IN HOSPITAL: APPOINTMENT OF SECRETARY.

Mr. E. Lionel Brown, of the Secretary-Superintendent's staff at the Middlesex Hospital, has been appointed Secretary of the City of London Lying-in Hospital, City Road. Mr. Brown who was in the London Rifle Brigade, has served in the war, been wounded, and retired from the Army; he entered the service of the Middlesex Hospital in 1910.

### WAR CHARITIES REGISTRATION BILL.

The inquiry made in our Note of July 29 in reference to the position of hospitals and other long-established institutions in the new war-charity legislation is answered in the text of the Bill introduced on behalf of the Home Office last week. The details of the proposed Act, which will come into operation one month after it becomes law, follow closely the lines of the Report of the Committee on War Charities, and are much as we foreshadowed. But what the institutional world was chiefly anxious to learn is the definition of a war charity, which the drafter of the Bill now determines as "any fund, institution, or association (whether established before or after the commencement of this Act) having for its object or amongst its objects the relief of suffering or distress, the supply of needs or comforts, or any other charitable purpose connected with the present war, but shall not include any fund, institution, or association established before the commencement of the present war where any such object as aforesaid is subsidiary only to the principal purposes of the charity." Any question whether a charity is a war charity will be finally determined by the Charity Commissioners. Evidently most of the voluntary hospitals taking wounded sailors and soldiers do not come within the Bill, but where a charity is using the whole of its establishment for this work, notwithstanding what its peace-time functions may be, it seems clear that, if voluntary contributions are solicited or received, registration must be sought.

### THE TUBERCULOUS SOLDIER.

At the annual meeting of the National Association for the Prevention of Consumption, held the other day at Hanover Square, W., Lord Balfour of Burleigh presiding, Sir William Osler read a thoughtful paper on "The Tuberculous Soldier." During the year 1915 no fewer than 2,770 soldiers were invalided from the Service on account of tuberculosis. In some cases, unfortunately, the disease may have been present in its earliest stages upon enlistment, whilst in others the physical disablement has been the direct outcome of military service. By an arrangement between the War Office and the National Insurance Commissioners, sanatorium treatment is now granted without delay to all tuberculous soldiers discharged from the Army, and, up to the present, the cases dealt with have amounted to 1,400. The question of an adequate pension for the men so disabled was, until recently, a matter for the gravest consideration, for, as has hitherto been the rule, the separation allowance ceases automatically when the tuberculous soldier is discharged from the Army, and the mental anxiety associated with this is hardly conducive to his recovery. It is gratifying, therefore, to record that Mr. H. J. Tennant announced at the meeting that men who joined the Army and were found to be tuberculous were to be awarded part of the pension—not the whole amount—if it could be proved that the disease had been aggravated by military service. This concession on the part of the War Office will go some way towards removing the heavy burden which the consumptive soldier and his family have had to bear. The suggestion made by Sir William Osler that doubtful cases of incipient or threatened phthisis occurring in recruits should be referred to the tuberculosis expert of the district strikes us as being most practical. The tuberculosis officer is on the spot, and he has the requisite experience for detecting the first beginning of the disease. Unless a man can pass the most rigid physical examination of the chest, he has certainly no right to be drafted into the Service even upon the dubious chance that he will be hardened by the training. The further recommendation that a national organisation should look after the welfare of the tuberculous soldier in co-operation with the after-care committees throughout the country will also meet with the approval of those who have the best interests of the men at heart.

### THE DETENTION OF TIME-EXPIRED DOCTORS.

At first sight the action of the War Office in refusing to let go a number of temporarily commissioned officers of the R.A.M.C. at the expiration of their contracts of service seems very harsh and arbitrary; but there are certain considerations which explain and to a great extent justify the action of the authorities. The message from Sir A. Keogh read to the British Medical Association meeting stated that the Order had been necessary on account of the small number of doctors who have recently volunteered: if this were the only justification of the measure, it would be difficult to defend the War Office authorities, for it is obviously their duty to watch the recruiting returns and to apply their compulsory powers in time to prevent shortage. Much more probably, though Sir A. Keogh does not say so, the refusal to call up doctors compulsorily—and the consequent necessity of retaining time-expired men in violation of their agreements—was due to the fear of giving away information to the enemy about the projected offensive. If the Germans had heard in June, let us say, that some hundreds of medical men were being hastily conscripted, the evidence of an impending British offensive on a large scale might have been more convincing to them than it would otherwise have been. If this was Sir A. Keogh's motive, we agree with his policy and applaud it. In any case, we have his assurance that the compulsory detention of the time-expired is temporary, and that the Order on the subject will be withdrawn as soon as a sufficient number of doctors are available. We disclose no secret when we say that a large proportion of the doctors of military age who have not hitherto served are now under notice to join up; and in a few weeks' time we imagine the Director-General will have such a profusion of the newly-commissioned that he will be able to let the compulsorily detained men go. It is hard lines on them in the meanwhile, but it cannot be helped; the interests of the Army must be the first consideration.

### A SMALL-POX HOSPITAL PROJECT AT EXETER.

An unfortunate *impasse* seems to have arisen at Exeter regarding the provision of a small-pox isolation hospital there, in anticipation of possible outbreaks of that disease after the war. It appears that a site has already been selected and bought; and the Sanitary Committee of the Exeter City Council has invited tenders for the erection of buildings, making of roads, and so forth, in accordance with plans drawn up by the City Surveyor. A tender of £1,215 for the erection of a four-bed pavilion has been recommended by the Sanitary Committee to the Council for adoption; but after a long debate the recommendation has been referred back by 21 votes to 19. It appears that several members of the Council felt that the Sanitary Committee should have consulted the Council before proceeding with the designs, or asking for tenders, in connection with this hospital. The site was bought, so it is said, with the idea of erecting temporary buildings on it in case an epidemic should arise, not for the purpose of a permanent building to be put up at once. The Finance Committee also feels hurt at not having been consulted over the raising of the necessary money. Furthermore, there is criticism of the site selected, though this is distinctly belated in view of the fact that it had been already bought some time ago. It is alleged that a healthier and better site could easily have been obtained near that which was chosen and at less expense.

### BOOKS FOR PRISONERS OF WAR.

The Board of Education has for a long time been paying close attention to the provision of literature for the British prisoners of war in foreign countries. Recent restrictions upon the transmission by private individuals of any printed matter to enemy or neutral countries makes it more than ever important that friends and correspondents of interned men, when writing to them, should make them acquainted with the existence of the Educational Book Scheme, by which they can get their wants supplied. Arrangements have been made whereby any prisoner can obtain, free of charge and carriage paid, good books of an educational character (not fiction or light literature) on almost any subject for reading or private study during his internment, by communicating with Mr. A. T. Davies, of the Board of Education, Whitehall. Prisoners are invited to state as precisely as possible on a form (which can be had *gratis* on application) what kind of books they desire. Offers of suitable books for this Educational Scheme will be gladly received by Mr. Davies; they should be accompanied by a list of the books proposed to be contributed.

### THE SURGICAL TUBERCULOSIS HOSPITAL AT NEWPORT.

As readers of The Hospital are already aware, there has been considerable opposition in the Clytha Park district of Newport to the proposal to establish there a hospital for surgical tuberculosis in children. The gift of Cardigan House and its site to the King Edward VII. National Memorial Association by Sir Garrod Thomas for this purpose is acknowledged by Mr. Bulwer Marsh, a Newport practitioner of forty years' standing in the profession, to be a piece of great generosity; but the acceptance of the gift is criticised as a grave blunder from the surgical point of view. Mr. Marsh and his fellow-residents in the Clytha Park district put themselves thoroughly in the wrong to begin with by basing their opposition on selfish and easily disposed of arguments; but they now make certain statements which improve their case considerably and demand investigation. He says the site and house have been condemned for the purpose in view by Mr. Robert Jones, of Liverpool, and Mr. Lynn Thomas, of Cardiff. Both these surgeons are of high authority on surgical tuberculosis; and if it is correct that the directors of the National Memorial Association asked them to report on Cardigan House and then acted flatly against the verdict, obviously explanations are due. Mr. Marsh enters in some detail, in a letter to the *Western Mail*, into the drawbacks of the site from an institutional point of view. If all his statements are well founded, Cardigan House cannot be described as ideal for the purpose it is to be put to. Possibly it was found to possess fewer disadvantages than any other available site; but a memorandum on the subject from the King Edward VII. Association would be reassuring, and seems to be called for.

### THE CORNER-STONE OF A HOSPITAL.

Notice has been given of a somewhat unusual topic for discussion at the monthly meeting of an Irish institution, the Cork Sanatorium. This is a copy of a resolution which has been received from the Kanturk Rural District Council, and runs as follows: "That before paying our contribution to the Joint Hospital Board, we would respectfully ask if a corner-stone in the sanatorium building at Streamhill states that the site was given as a free grant by Mr. Creagh, notwithstanding that the ratepayers have paid a sum of £400 quite recently for the same site, and if any steps will be taken to remove this farcical inscription." Whether or not there is any foundation for the allegations brought by the Kanturk Council, in either event the incident has an amusing side; unless the deadly seriousness of the Kanturk Council is a subtle form of Irish wit, we should feel inclined to say that they are the only people who cannot see the humorous side of their resolution.

### THE FUTURE OF BRUNSWICK HOUSE.

Brunswick House, Mistley, which is at present used as an annexe to Colney Hatch Mental Hospital to shelter thirty-seven chronic male lunatics, is now to be used for the reception of cases of mental defect. The London County Council has experienced very great difficulty in finding accommodation for male defectives, principally between the ages of sixteen and twenty-one. There is at present no certified institution within the Council's knowledge to which these cases can be removed. The only beds at present available to the Council are those provided in the Woolwich Guardians' workhouse, but this accommodation is limited to twenty-five vacancies, and it is rightly felt that it would not be desirable to send to a workhouse young lads susceptible of improvement. At Brunswick House it will be possible to find room for more than fifty cases. The present equipment of the institution will be available, and the Board of Control have intimated their sympathy with the proposal for the use of the property for this new purpose.

### ANOTHER EXTENSION AT COVENTRY HOSPITAL.

The committee of the Coventry and Warwickshire Hospital has been requested by Colonel Marsh to provide another twenty-two beds for wounded soldiers. A scheme, therefore, is being proceeded with, and a good start has been given by a donation of £500 from Messrs. Iliffe and Sons, a firm to which the hospital already is indebted for much. There happens to be situated next door to the out-patient department Sir Thomas White's School, and the trustees have allowed the hospital authorities to take it over. The cost of fitting the school for hospital purposes will be £2,000, but when this has been done no fewer than 160 wounded soldiers will be accommodated at the hospital. The general policy of adapting schools in this way is one which we have often criticised; and while we are still averse to their use in principle, the convenience of the school's situation in the present case is not to be denied.

### THE SCOTCH "STAR AND GARTER."

The Ralston Hospital, situated between Glasgow and Paisley, has been opened by the Duchess of Montrose, and has a special interest from being the Scotch counterpart of the "Star and Garter" Hospital at Richmond, established for paralysed and permanently disabled soldiers. The building is like the "Star and Garter" in the sense that it has a fine situation, if not a unique view; and the rooms and corridors are spacious, airy, and well lighted. In 1890 the house was bought by Sir Charles Cayzer, Bart., who, after equipping it for its new purpose, has given the free use of the house for a period of ten years as a hospital for paralysed soldiers. It is hoped that the institution, which comprises twelve wards and accommodates almost eighty patients, will prove sufficient to meet the needs of all Scotch soldiers suffering from paralysis. The equipment necessary has extended not only to the interior of the building but to the grounds, which have been provided with gangways to allow the ready access of bedridden patients to the lawn and suitable corners of the gardens. Hitherto the Ralston Hospital has been occupied chiefly by convalescents.

### THE STORE ON THE TERRACE.

When the military huts for wounded soldiers were opened at St. Thomas's Hospital in June 1915 the ladies of the medical staff opened a fund "to provide additional comfort and brightness for the patients," which has now published a brief report of its first year of work. Some £677 was collected at the original meeting, of which a considerable sum was spent on furniture for the huts and recreation-rooms. Then a store-room was established on the terrace, which was run on the following lines. Tobacco, writing materials, and groceries were kept there, and served by relays of ladies on three days a week. Each hut and ward sent down a list of its requirements based upon the number of its occupied beds. In time, however, the fund grew smaller, and gradually biscuits and sweets were given up and the allowance per head of cigarettes and matches grew smaller. Even pipes and pouches, which had been given as presents, were sold at half-price in the recreation-room. The fund has now been practically exhausted, and if anyone wants an additional reason for subscribing to the work he will surely find it in the administrator's statement, that in his opinion "the steadiness of the men and the absence of breaches of discipline are mainly due to the consciousness of the soldier that his comfort and interest are looked after by various voluntary agencies" like this fund.

Mrs. Hawkins, 56 Portland Place, and Mrs. Battle, 49 Harley Street, will gladly receive gifts of money or jam, pickles, stationery, matches, soap, and woodbines!.

### THE WELSH MEDICAL SCHOOL DELAY.

The building of the Welsh National Medical School at Cardiff has been arrested by the refusal of the Ministry of Munitions to allow the medical research block to be commenced, on the ground that the large amount of labour and constructional steel required on it cannot be now employed without detriment to the national interest. No objection is raised to the continuance of work on the physiological wing, which is nearing completion. As this unlooked-for delay is likely to impair greatly the immediate usefulness of the medical school, and militate against its success in many ways, energetic efforts are being put forth with the object of getting the embargo removed. It is pointed out that all the labour employed on the building is ineligible for military service, and that the constructional steel and all the other necessary material are in hand at Cardiff and awaiting use.

### AN APPEAL TO THE WORKERS OF CARDIFF.

A special appeal to the working classes for a still more generous support of the institution was made at a meeting in connection with the King Edward VII. Hospital at Cardiff. Colonel Bruce Vaughan pointed out that the hospital would shortly require an additional £10,000 a year income, and he remarked that the number of waiting patients, viz. 1,013, was an ample excuse for their appeals for greater support. Mr. Leonard Rea, secretary of the hospital, said that Leicester received £15,000 a year from the working men, and Newcastle-on-Tyne £23,000, while Cardiff only got £8,000. If all the collieries in the district adopted a penny-a-week system, the King Edward VII. Hospital would have almost as large a sum as the Newcastle Hospital.

### MUNIFICENT BEQUESTS TO GLASGOW CHARITIES.

Among the charitable bequests of Mrs. L. M. Craig, wife of a Glasgow merchant, to become operative on the death of her husband, are the following: £2,000 to the Royal Cancer Hospital, Glasgow; £1,000 each to the Eastpark Home for Infirm Children, Glasgow, the Orphan Homes of Scotland, Royal Hospital for Sick Children, Glasgow, the Western Infirmary, Glasgow, (to endow a bed), and Dr. Barnardo's Homes; £500 each to Miss Annie Croall's Children's Home, Stirling, the Glasgow and West of Scotland Convalescent Seaside Homes, Dunoon, Kilmun Convalescent Seaside Homes, Old Women's Home, Glasgow, Royal Infirmary, Glasgow, Royal Samaritan Hospital for Women, Glasgow, and Victoria Infirmary, Glasgow; and £250 to the Samaritan Society connected with the Western Infirmary, Glasgow.

### COMPLAINTS AT A STAFFORDSHIRE SANATORIUM.

An investigation has been conducted into the cause of so many patients sent to Staffordshire tuberculosis sanatoria taking their discharges. From a report presented to the County Insurance Committee it appears that six cases left owing to domestic circumstances and nine because they said they felt better. In four cases, however, the reason for the patients leaving was the alleged poor quality of the food supplied, and in five cases the complaints were of a miscellaneous character. With regard to the food, the committee is satisfied that there was either no grounds for complaint or that the grievances have been remedied, but two of the miscellaneous complaints are looked upon as serious and are to be inquired into further.

### TWO NEW COURSES OF TRAINING FOR WOMEN.

With all the new careers that are opening to women, both as the result of the war and by the development of various sorts of social advancement, there is undoubtedly some risk of pitchforking untrained persons into employments where they may fail in efficiency for lack of suitable instruction beforehand. Just recently two new courses of training have been devised, both of which seem admirably adapted for their respective purposes. The Royal Sanitary Institute has issued a syllabus of an examination for maternity and child-welfare workers: it is in sequence with the existing examinations for health visitors and inspectors of nuisances, and those who have passed it are bound to receive preferential treatment from local authorities when selecting candidates for appointments to posts under infant-welfare schemes. The other syllabus is one drawn up by the Royal Institute of Public Health of a course of instruction for women in chemical, bacteriological, and pathological laboratory work. This kind of skilled labour has been until recently entirely in the hands of men; but it is of such a light kind physically as to be within the capacity of almost any woman. At the end of the course, which lasts three months, an examination will be held and certificates of competency given. The course should result in a great lightening of institutional difficulties owing to the absence of "lab. boys" in pathological departments.

### MILITARY HOSPITALS AND UNNECESSARY TELEGRAMS.

The secretary of the semi-military hospital, the A B C of whose education teaches that unnecessary administrative expense is almost a crime, has now somewhat lost his early feeling of astonishment at the lavishness of military authorities in the matter of telegrams. When the telegram costs nothing to the sender and conveys as effectual a message as would a letter the taxpayer alone suffers, but when the wording is careless and causes correspondence and delay there is more to be said. An instructive example of the condition last described recently occurred at a London hospital. A telegram signed "Thundergun, Muddleton," requested confidential information to be telegraphed about a military patient. As, obviously, the secretary could not answer an anonymous and possibly unauthorised inquiry no answer was sent. A week elapsed and another telegram arrived in exactly the same words. Again no reply was sent, and after another week a letter came to the effect that "The Officer Commanding the Royal Garrison Artillery, Muddleton, wishes to know when 11784 Gunner Smith, R.G.A., etc., etc. It is not understood by the O.C. why no reply was sent to the communications of June 7 and 14." The secretary's reply was as follows: "In answer to the inquiry of the O.C. the secretary begs to state that 11784 Gunner Smith, R.G.A., was . . . It is not understood by the secretary why the O.C. should expect him to communicate information of a private nature in response to an anonymous inquiry, and why a charitable institution should be asked to expend money upon a telegram when the necessary information can be supplied, as it could be solicited, by letter."

### QUAKERS' RELIEF WORK IN RUSSIA.

The Society of Friends has lately been to the fore in providing a kind of stock test as to the sincerity of conscientious objectors, but it has also been engaged in hospital and relief work of its own. As a result of a deputation, sent out to Russia by the Society of Friends' War Victims' Relief Committee in the spring, a relief party, consisting of four doctors with a staff of eight men and ten women, has left Newcastle for Russia. The deputation was specially commended to visit the country bordering the Volga, and in the Samara district were offered by the Governor a hospital, hitherto unoccupied through the war, with accommodation for 200 cases. It is estimated that the number of refugees now in Russia is two and a-half million, and their principal needs appear to consist in medical attendance, nursing, and a supply of clothes. There exists in Russia an organisation known as the United Relief Committees, from which it would appear that the country has been mapped out so as to apply the relief organisations to the different districts as required.

### A TEMPORARY WARD BURNT TO THE GROUND.

One of the few instances of fires in temporary hospital buildings occurred last week at the King's Cross Fever Hospital, Dundee, where one ward was burnt to the ground and another considerably damaged. At about 10.20 in the morning the gardener noticed smoke issuing from the wooden framework of one of the windows in Ward 7, which is luckily detached from the main block by about 20 yards, and was built to relieve the pressure on the other wards. Fortunately there were no patients in the ward at the time, but it contained thirty beds and a considerable quantity of bedding. Acting with praiseworthy promptitude, the gardener, Mr. David Bell, tried to subdue the flames with the hospital hose, but the ward, made of corrugated iron lined with wood and felt, blazed fiercely, aided by a strong wind, so that even when the fire brigade arrived they could do little more than prevent the flames from spreading. The nearest part of the main buildings is Ward 5, which took fire in several places, including the adjoining bathroom. In this ward were five children and two adults, whom the matron, Miss Clark, had promptly removed, together with the movable furniture. The collapse of the roof of No. 7 Ward proved the limit of the conflagration, though local outbreaks had to be controlled as its result. The fire is supposed to have been caused by the heat of the sun on the corrugated-iron roof causing the wood or felt beneath to catch alight. The damage is estimated at some £2,500.

### HOSPITAL ATTENDANTS KILLED IN ACTION.

The Visiting Committee of the Graylingwell Mental Hospital, formerly known as the West Sussex County Institution, has issued an interesting survey of the effects of the War Office occupation. When it was taken over on April 1, 1915, the original patients were mostly distributed among ten other mental hospitals, but the staff was transferred with Dr. Kidd, the medical superintendent, acting as Administrator of the War Hospital, with the rank of Lieutenant-Colonel. With the financial arrangements, elaborated on the usual lines, we need not deal, but a special reference is due to the patriotic action of the attendants. Those who were on the Reserve at the outbreak of war joined the Colours, and later practically the whole of the male indoor staff enlisted in the Royal Army Medical Corps and remained at the hospital. Then those of the outdoor staff who were eligible attested under the Derby Scheme, and a few who were considered indispensable by the Administrator have received exemption or postponement. Four of the attendants have been killed on active service. The committee is appealing for funds to place a memorial in Chichester Cemetery to soldiers who die at Graylingwell as a result of sickness or wounds inflicted during the war. We hope, too, that the claims of the attendants will not be forgotten, and that a suitable memorial will be promptly placed in the institution to those of the staff who have died on active service. No one has a higher claim, and our great mental hospitals should be quick to recognise it.

### DEGRADATION IN MONMOUTHSHIRE.

Acting on the advice of their chief medical officer, Dr. Rocyn Jones, the Monmouthshire County Council last week resolved to establish twenty Maternity and Infant Consultation Centres, at a capital cost of £400 and a maintenance expense of £1,450 a year. The need of such a scheme is apparent from the medical officer's annual report, which discloses an appalling state of affairs in the county. In the first place, Dr. Rocyn Jones points out that practically 600 babies, out of the total of 9,459 last year, were still-born. A special investigation is being conducted into this matter. In some of the industrial towns mothers of babies in arms were found under the influence of drink at 11 o'clock in the morning. The compulsory notification of births had revealed the existence of a large number of hovels of which there had been no knowledge previously. Cellars and underground tenements were found occupied by a number of families, who used the single apartment as living and sleeping room. Babies were sleeping with persons suffering from consumption. The nurses'

reports showed absolute ignorance as to child welfare on the part of young mothers in both industrial districts and agricultural areas, and the infant mortality would have been far higher but for the work of the county lady health visitors. The mortality figure goes up to 193.5 in the urban district of Caerleon and 115 in the rural district of St. Mellons. Dr. Rocyn Jones mentions that unregistered midwives had not hesitated to take a defiant attitude towards the health visitors, and in some instances had attempted to undermine the advice given.

### THE SPIRITUALISTS' AMBULANCE.

MANY organisations have contributed ambulances to our Armies and those of our Allies, but perhaps the most curious is a car with an inscription relating that it has been "jointly subscribed by the Spiritualists of the Dominion of New Zealand and Great Britain." In fact, the fund raised by them has provided six cars in all. We understand that March 31 last, when the first portion of the work undertaken by the fund was completed, was the sixty-eighth anniversary of modern Spiritualism. Exactly what this implies is not apparent to those outside the Spiritualist movement; but the generosity of the Spiritualists cannot be questioned, and the cars they have given are on service, we understand, both in Egypt and France.

### SUBSTITUTES FOR COCAINE.

A GOOD deal of unnecessary and ill-informed fuss has been made, since the Cocaine Regulations were issued, concerning the hardships inflicted on unregistered dentists by the prohibitions contained in the Regulations. The result is that as a temporary measure permission has been granted to such dentists to procure cocaine up to September 15 in one-per-cent. solution, subject to the condition that it shall be used solely as a local anæsthetic. One member of Parliament stated his belief that no fatalities had ever attended the use of cocaine by unregistered dentists. This statement is quite untrue, it is hardly necessary to say; chapter and verse can be given if necessary. And serious consequences, short of actual death, have been quite common when cocaine has been used by unregistered dentists. In point of fact, eucaine and novocain are so well adapted in every way for dental analgesia that it would be no bad thing if cocaine could be prohibited altogether, even for registered dentists, and *a priori* for the unregistered. The amazing ignorance of public men about these drugs is illustrated, not only by the remarks of an M.P. mentioned already, but also by those attributed in the Press to a well-known London Coroner, who informed his jury that stovaine is a derivative of cocaine, which is not the case.

### THE WALKER-GORDON MILK LABORATORIES.

AN unfortunate consequence of the war has been the closing down, temporarily at least, of this well-known firm of clean-milk purveyors. The handling of milk so as to ensure the nearest approach to freedom from contamination, the preparation of milk prescriptions, and similar facilities for obtaining pure milk for infants which this firm was one of the first in Great Britain to provide, require the services of expert employees who cannot be easily or hurriedly replaced. So many of them have joined the Forces that, combined with other war difficulties, the work of the dairies and laboratories has for some time been carried on at a loss. It has therefore been decided to close down until the war is over. The reputation which this firm enjoys will, we trust, ensure the resumption of business when the exceptional circumstances of the present time have passed away; for London can ill afford to lose permanently any of the factors which make for improvement in the quality of the milk supply, and the Walker-Gordon Laboratories have an honourable record in this respect.

### THE AUGUST "SOUTHERN CROSS."

THE August number of the *Southern Cross*, the monthly gazette of the 1st Southern General Hospital, Birmingham, is chiefly filled with humorous sketches and light verse, mainly of local interest. We notice, too, that a hint has apparently been taken from the lively pages of *Truth*, for "S. C. A.," disguised as the shade of the great diarist, has contributed a two-page article entitled "A Peep Behind the Scenes." The column "News from Old Friends" should make an agreeable link between those who have left and their friends still in hospital, especially if it can be developed. One of the Scotch hospital gazettes has made quite a feature of interesting articles by former members of its staff or patients now serving in other parts of the world. We mention this to remind the editor of the *Southern Cross* that such articles, seemingly so hard to secure, can yet be come by, and the magazine which contains them is not so quickly thrown aside as those that aim at merely comic effects.

### THIS WEEK'S DRUG MARKET.

IT is difficult to see how, in face of the greatly curtailed demand for cocaine, the present price can be maintained; although the stock of cocaine in the country is small, it will still be possible to import supplies under licence, and, considering all the circumstances, it is not improbable that the price, which was not immediately affected by the new regulations, will tend downwards; in any case, cocaine is not a drug which it would be advisable to buy at the present moment. As to opium, the general opinion is that prices will be affected but little by the new regulations for the present. The drug market has continued quiet, and the general tendency of prices is still downwards. An exception is to be found in the case of Japanese camphor, which has advanced in price considerably and appears likely to advance still further; so far, English refiners of camphor have not raised their quotations. Tartaric acid and citric acid are both lower in price. Salicylic acid and salicylate of soda continue to tend downwards in value. Acetylsalicylic acid and phenazone have a lower-price tendency. Bromides are quiet and unchanged.

